# Inter-individual differences in working memory improvement after acute mild and moderate aerobic exercise

**DOI:** 10.1371/journal.pone.0210053

**Published:** 2018-12-31

**Authors:** Yudai Yamazaki, Daisuke Sato, Koya Yamashiro, Atsuhiro Tsubaki, Nana Takehara, Yoshihito Uetake, Saki Nakano, Atsuo Maruyama

**Affiliations:** 1 Institute for Human Movement and Medical Sciences, Niigata University of Health and Welfare, Niigata, Japan; 2 Graduate School for Major in Health Science, Niigata University of Health and Welfare, Niigata, Japan; 3 Department of Health and Sports, Niigata University of Health and Welfare, Niigata, Japan; 4 Department of Physical Therapy, Niigata University of Health and Welfare, Niigata, Japan; Iwate Medical University, JAPAN

## Abstract

Many studies have shown that aerobic exercise improves cognitive function and maintains brain health. In particular, moderate-intensity exercise is effective for improving cognitive performance. However, there is no strong consensus on whether a single exercise session improves working memory (WM) function, as it does inhibitory function. It is possible that these discrepancies involve inter-individual differences in WM function. Therefore, we investigated whether acute mild and moderate aerobic exercise improve WM, and whether there exist inter-individual differences in improvements in WM. Thirty healthy subjects were recruited and participated in three experimental conditions (control, mild-intensity exercise, and moderate-intensity exercise). Subjects performed 10 min of exercise on a cycle ergometer with an individualized load. Their pedaling rate was maintained at 60 rpm. In the control condition, subjects rested on the cycle ergometer instead of performing exercise. The N-back task (2-back and 0-back task) was performed to assess WM function before, 5 min, and 15 min after the 10-min exercise session. In this study, to elucidate the effect of an acute bout of mild or moderate exercise on WM, the “2-back– 0-back” contrast, which is assumed to represent WM function, was calculated. The Two-Dimensional Mood Scale was adopted to measure changes in psychological mood states efficiently. The results revealed that working memory function was not improved by acute mild or moderate exercise. However, baseline working memory function was significantly associated with any change in working memory function following exercise, and this was independent of exercise intensity. Subjects with the lowest working memory function at baseline responded the most favorably. The results revealed that improvements in working memory function after a single session of aerobic exercise depend on baseline working memory function.

## Introduction

“Executive functions” are mental processes involved in purposeful thinking and goal achievement, and are regulated by the prefrontal cortex [1]. These functions comprise three subcomponents: inhibition, working memory (WM), and cognitive flexibility [2]. These higher-order brain functions facilitate more complex mental activities such as reasoning, planning, and problem-solving. One of the executive function subcomponents, WM refers to the temporary storage and manipulation of information necessary for complex tasks such as language comprehension, learning, and reasoning [3].

WM requires necessary information to be maintained in limited capacity, and unnecessary information to be inhibited. In addition, the information that is stored in WM will be reconstructed, depending on purpose and situation. The function of updating is the need to reconstruct information in WM storage. Updating is defined as the modification of necessary information [4]; in other words, new information is added into WM storage, and irrelevant or unnecessary older information is removed from WM storage. Updating is associated with complex cognitive function, such as fluid and crystallized intelligence [5], reading comprehension [6], and arithmetic calculations [7]. Therefore, keeping a high level of WM function, including updating, is important to prevent the decline of cognitive function and to execute cognitive processing properly.

Many studies have shown that aerobic exercise improves cognitive function and maintains brain health [8–11]. Voss et al. [12] reported that a 1-year regime of aerobic exercise increased functional brain connectivity (e.g., default mode network and frontal executive network) and improved the brain’s ability to perform executive functions. Moreover, physical activity is associated with memory encoding and gray matter volume in the prefrontal and cingulate cortices [13]. Interestingly, even a single session of exercise has been reported to enhance learning, memory, and cognitive function [14–16]. Regarding WM, a previous study using meta-analysis demonstrated that acute moderate-intensity aerobic exercise facilitates the speed of processing in a WM task, and that the effect size was low to moderate [17]. In addition, Roig et al. (2013) indicated that the influence of acute moderate-intensity aerobic exercise on short-term memory and WM had a more than moderate effect [15]. However, previous studies that assessed the effect of acute aerobic exercise on WM via an updating task have showed inconsistent results. Therefore, it remains unclear what the influence of acute aerobic exercise is on WM and updating. Pontifex et al. (2009) and Weng et al. (2015) have reported that acute moderate-intensity aerobic exercise improves the accuracy of a modified Sternberg test or N-back task [18, 19]. Conversely, Gothe et al. (2013) and Li et al. (2014) have demonstrated that reaction time and accuracy in an N-back task were not improved by acute moderate-intensity aerobic exercise [20, 21]. It is possible that these discrepancies involve inter-individual differences in WM function as described by Sibley and Beilock [22]; they indicated that there is individual difference in the improvement of reading and listening span tests by acute moderate-intensity aerobic exercise, and reported that the low-performance group at baseline improved task performance after exercise. While reading and listening span tests can assess WM capacity, an N-back task can measure the updating function. It is thus not known whether individual differences occur, regarding the influence of acute aerobic exercise on updating function. In addition, previous studies that assessed an updating task only used moderate-intensity exercise; the effect of acute low-intensity aerobic exercise on WM and updating is unknown. In the present study, we investigated whether moderate and mild-intensity exercise improves WM updating function, and whether inter-individual differences in updating function are involved in the effect of acute exercise on WM, irrespective of exercise intensity.

We hypothesized (a) that acute aerobic exercise would improve updating performance and that moderate-intensity exercise would be more effective; (b) that this improvement would have high inter-individual variability; and (c) that this variability would depend on baseline performance, irrespective of exercise intensity. In other words, subjects with higher performance at baseline assessment would be less affected by the exercise, while subjects with lower performance at baseline would be more affected by the exercise.

## Materials and methods

### Participants

We enrolled 30 healthy volunteers aged 19–31 years (11 females, age = 21.8±1.7 years). All subjects were right-handed, none had a history of neurological or psychiatric disease, and none were taking any medications at the time of the study. Informed consent was obtained verbally from all participants, and the present study was conducted in accordance with the Declaration of Helsinki and with the approval of the ethics committee of Niigata University of Health and Welfare.

### Experimental procedure

**Fig 1:**
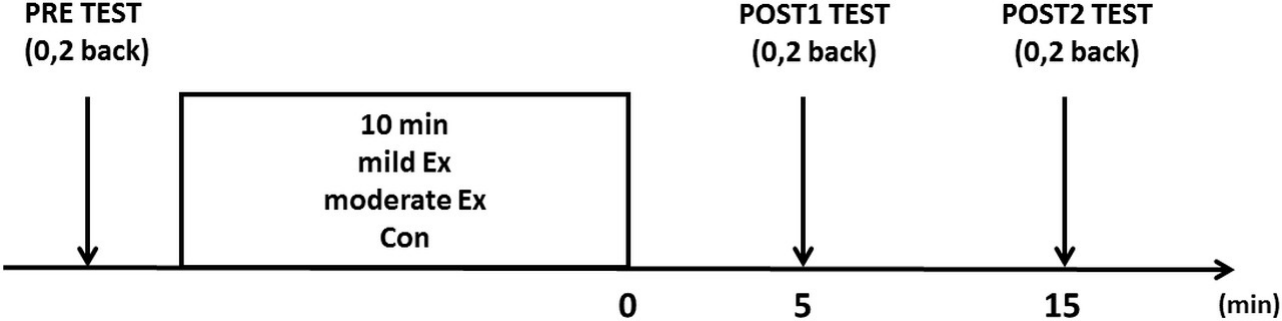
Experimental protocol. N-back tasks (0-back and 2-back) were performed before, 5 min, and 15 min after the intervention.

The overall procedures consisted of two stages: preliminary and main experiments. First, maximal oxygen uptake (${\dot{V}O}_{2\mathrm{peak}}$) was measured to determine the appropriate individual intensity for mild and moderate exercise, which was defined as 30% and 50% of a subject’s ${\dot{V}O}_{2\mathrm{peak}}$ based on the classification of physical activity intensity by the American College of Sports Medicine [23]. Second, three main sessions including 1) control (CON), 2) mild exercise (mild Ex), and 3) moderate exercise (moderate Ex) were conducted, according to a randomized design on different days at least 5 days apart. All subjects participated in each experiment, with the order in which they performed each session being randomized, as shown in Fig 1. In both Ex sessions, the participants conducted the N-back tasks in the order of 0-back followed by 2-back, and performed them before, 5 min, and 15 min after the exercise session. This is because non-cortically derived physiological parameters are known to increase with 10 min of exercise at mild and moderate intensities, and return to basal levels within 5 min and 15 min, respectively [24, 25]. Subjects performed 10 min of exercise on a cycle ergometer with an individualized load. Their pedaling rate was maintained at 60 rpm. In the CON session, subjects rested on the cycle ergometer instead of performing exercise. A psychological mood state scale, described below, was completed before each N-back task. Rating of perceived exertion (RPE) was assessed using a Borg’s scale, ranging from 6 (no exertion at all) to 20 (maximal exertion) after each exercise [26].

### Cardiovascular test

Prior to the main experiments, a graded maximal exercise test was performed on the cycle ergometer to determine a subject’s ${\dot{V}O}_{2\mathrm{peak}}$, using a method modified from previous studies [24, 25]. Mild and moderate exercise intensity were defined as 30% and 50% of a subject’s ${\dot{V}O}_{2\mathrm{peak}}$, based on the classification of physical activity intensity by the American College of Sports Medicine. Exercise began with 1 min of warm-up at 60 W and increased 20 W (female: 15 W) every 1 min until exhaustion. Subjects were instructed to maintain a pedaling rate of 60 rpm. Heart rate was measured by using a polar heart rate monitor (CS400, Polar Electro Oy, Finland) and was recorded every 1 min. In addition, RPE was verbally reported every 1 min. Ventilation parameters, oxygen intake (${\dot{V}O}_{2}$), and carbon dioxide output (${\dot{V}\mathrm{CO}}_{2}$) were measured breath-by-breath using a gas analyzer (Aeromonitor AE300, Minato Medical Science, Osaka, Japan) at a sampling rate of 0.1 Hz [27]. ${\dot{V}O}_{2\mathrm{peak}}$ was determined when two of the following criteria were satisfied: R > 1.15, achievement of age-predicted peak heart rate (HR peak), and an RPE of 19 or 20 [24, 25]. The exercise intensities of 30% and 50% ${\dot{V}O}_{2\mathrm{peak}}$ were calculated from the linear regression between ${\dot{V}O}_{2}$ and output power of ${\dot{V}O}_{2\mathrm{peak}}$ [28]. Subjects’ ${\dot{V}O}_{2\mathrm{peak}}$ and other respiratory and metabolic parameters are shown in Table 1.

**Table 1:** Subjects’ characteristics.

	HR (bpm)	RPE	RQ	Workload (kp)	${\dot{V}O}_{2}$ (ml/kg/min)
${\dot{V}O}_{2\mathrm{peak}}$	180.8±10.9	19.4±0.9	1.3±0.1	3.1±0.5	36.4±4.5
50% ${\dot{V}O}_{2\mathrm{peak}}$	130.5±7.7	13.4±1.4	1.1±0.1	1.6±0.3	20.2±2.6
30% ${\dot{V}O}_{2\mathrm{peak}}$	110.1±7.2	10.9±1.6	1.0±0.1	1.1±0.3	14.5±2.4

Mean values of heart rate (HR), rating of perceived exertion (RPE), respiratory quotient (RQ), workload (kp), and oxygen intake (${\dot{V}O}_{2}$) recorded at the end of exercise at intensity of ${\dot{V}O}_{2\mathrm{peak}}$ and estimated 30% and 50% ${\dot{V}O}_{2\mathrm{peak}}$ for each subject (mean±SD).

### Psychological measures

In the present study, we assessed the subjects’ arousal and pleasure levels by using the Two-Dimensional Mood Scale (TDMS), which was adapted to measure the changes in psychological mood states in an efficient manner [25, 29]. TDMS consists of 8 words: energetic, lively, lethargic, listless, relaxed, calm, irritated, and nervous. Subjects reported how closely their feelings matched these items from 0 (not at all) to 5 (extremely). TDMS was carried out before each N-back task, and each subject’s arousal and pleasure levels were calculated using these 8 words.

### WM measures

**Fig 2:**
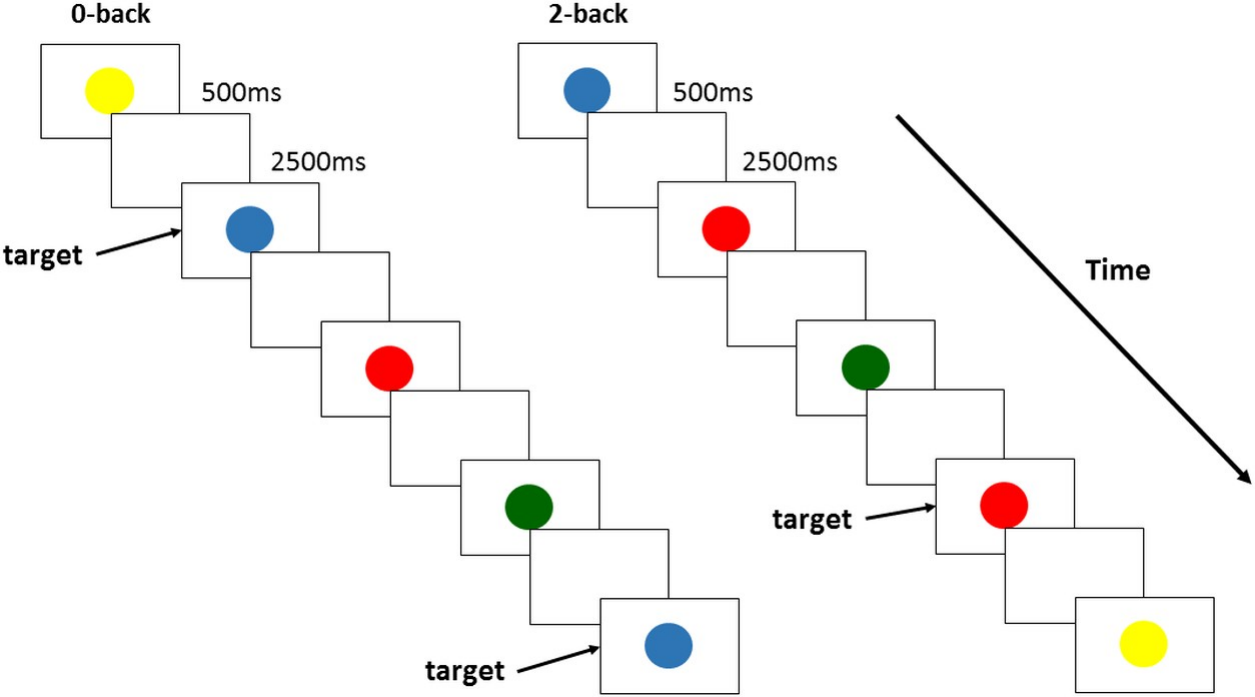
N-back task protocol.

WM updating performance was assessed with 2-back and 0-back tasks using four colored circles (red, green, blue, or yellow). N-back tasks were performed in the quiet laboratory. Stimuli were presented on a computer screen and were controlled by SuperLab software (Cedrus, CA). In the 2-back task, participants were presented with a continuous sequence of colored circles and instructed to detect whether each color matched the color presented two circles back in the sequence (Fig 2). In the 0-back task, the participants had to decide whether the color they saw on the screen was an instructed color. The target color was verbally instructed by the experimenter before starting each 0-back task. In the 0-back condition, the participants required sustained attention but not WM demand, including maintenance or updating of information [30, 31]. Each circle was presented for 500 ms and separated by a 2500 ms interstimulus delay, requiring successful encoding, maintenance, and retrieval for successful performance. If the current color and N steps earlier stimulus were matched, participants were required to push the “○” button with the left index finger, and if mismatched, to push the “×” button with the right index finger using a response pad. Each block condition included 30 stimuli presented in a random order. The reaction time (RT) and error rate of each participant were recorded. Prior to the experiment, the participants performed three practice blocks of each task.

In the present study, to elucidate the effect of an acute bout of mild or moderate exercise on updating function, the 2-back– 0-back RT contrast was calculated.

### Statistical analysis

HR, each N-back task RT, error rate, and 2-back– 0-back RT contrast were analyzed using a repeated-measures two-way (session × time) analysis of variance (ANOVA) (IBM SPSS Version 18, IBM, USA). To investigate the relationship between the baseline performance and exercise effect, the 30 subjects were divided into two groups high performance at baseline and low performance at baseline according to median value. A three-way repeated-measures ANOVA with the main factors being session (moderate Ex, mild Ex, and CON), time (PRE, POST1, and POST2), and baseline performance (high performance and low performance) was conducted. If the assumption of sphericity was violated in Mauchly’s sphericity test, the degree of freedom was corrected using Greenhouse-Geisser correction coefficient epsilon, and the *F*- and *P*-values were then recalculated. When the main effects were identified, a Bonferroni post-hoc multiple-comparison test of significant difference was performed to identify the specific difference in factors contributing to the variance observed in the data. The data relating to exercise intensity (RPE) immediately after exercise were analyzed using a paired *t*-test. To examine the relationships between baseline performance and the variation of performance from pre-exercise to post-exercise, Pearson’s product-moment correlation coefficient was calculated. The significance level was set at *P* < 0.05. Power analyses indicated that a total of 26 participants would be needed to detect an effect size *F* of 0.5, with two-sided alpha set at 0.05 and power at 0.8.

## Results

### Exercise intensities

At the end of moderate-intensity exercise, the average HR and RPE were 146.1±14.3 and 12.9±1.8, respectively. At the end of mild-intensity exercise, the average HR and RPE were 121.3±14.2 and 10.5±1.7, respectively. The results of the paired *t*-test revealed that RPE were significantly higher for moderate-intensity exercise than for mild-intensity exercise (*t* (29) = 5.992, *P* < 0.001). A two-way repeated-measures ANOVA showed that there was a significant main effect of session (*F*_(2,58)_ = 117.106, *P* < 0.001), time (*F*_(1.483,42.997)_ = 586.848, *P* < 0.001), and their interaction (*F*_(3.421,99.202)_ = 303.267, *P* < 0.001). A post-hoc test revealed that HR was significantly increased after exercise (moderate Ex: *P* < 0.001, mild Ex: *P* < 0.001), and it returned 5 min after exercise. In addition, HR after exercise in the moderate Ex condition was significantly higher than that of mild Ex (*P* < 0.001).

### WM performance

**Fig 3:**
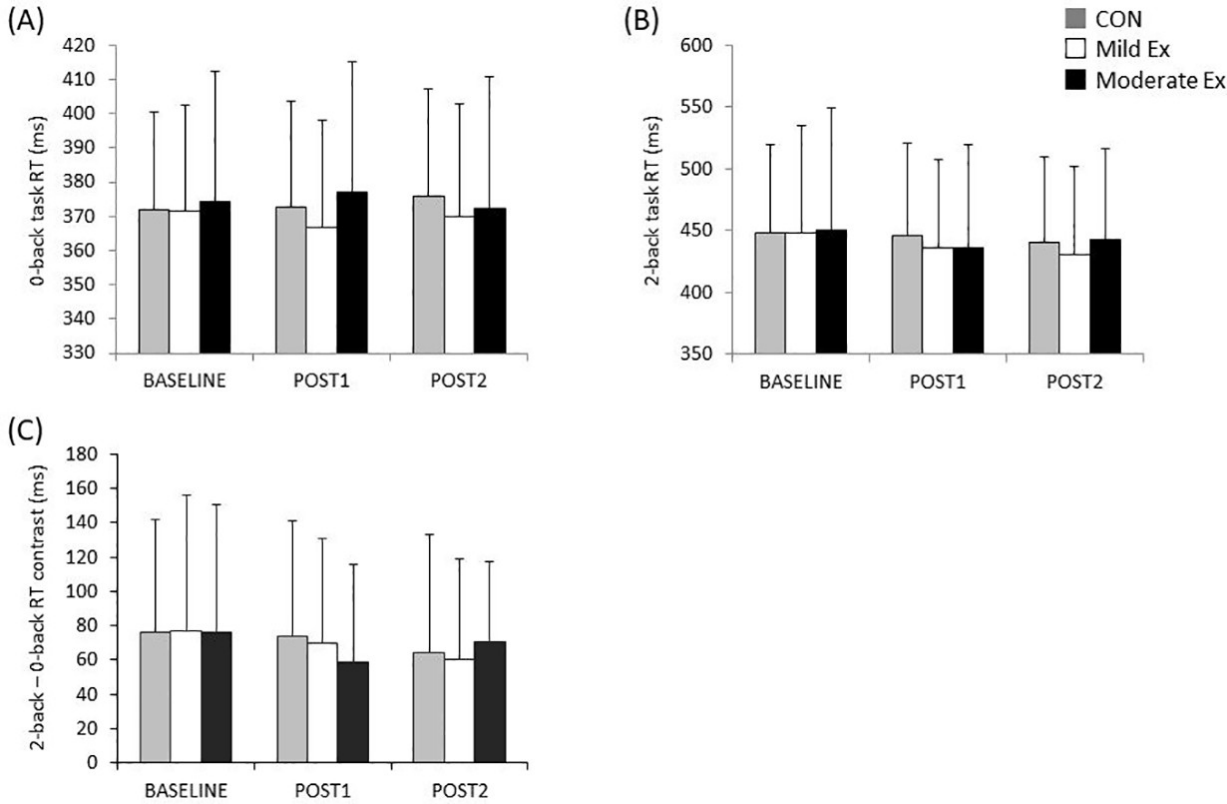
Changes in RT and WM function of each session. This figure shows the changes in RT and 2-back– 0-back RT contrast for each session: (A): 0-back task RT, (B): 2-back task RT, and (C): 2-back– 0-back RT contrast. The gray, white, and black bars indicate CON, mild Ex, and moderate Ex sessions, respectively. The error bar indicates standard deviation (SD).

Fig 3 shows the changes in 0-back RT, 2-back RT, and 2-back RT– 0-back RT contrast with each session. A two-way repeated-measures ANOVA revealed that there were no significant main effects of session (*F*_(2,58)_ = 0.428, *P* = 0.654), time (*F*_(2,58)_ = 0.041, *P* = 0.959), and their interaction (*F*_(3.050,88.451)_ = 0.850, *P* = 0.472) in the 0-back RT. Similarly, there were no significant main effects of session (*F*_(2,58)_ = 0.096, *P* = 0.908), time (*F*_(2,58)_ = 3.437, *P* = 0.059), and their interaction (*F*_(2.878,83.463)_ = 0.719, *P* = 0.538) on the 2-back RT. Regarding 2-back– 0-back RT contrast, a two-way repeated-measures ANOVA showed that there were no significant main effects of session (*F*_(2,58)_ = 0.031, *P* = 0.970), time (*F*_(2,58)_ = 2.863, *P* = 0.065), and their interaction (*F*_(2.760,80.043) =_ 0.875, *P*_=_ 0.450).

Regarding error rate, a two-way repeated-measures ANOVA revealed that there were no significant effects of session (*F*_(1.321,35.661)_ = 0.846, *P* = 0.394), time (*F*_(2,58)_ = 2.575, *P*_=_ 0.085), and their interaction (*F*_(2.626,70.193)_ = 0.540, *P* = 0.633) on the 0-back task. Similarly, a two-way repeated-measures ANOVA showed that there were no significant effects of session (*F*_(2,38)_ = 0.842, *P* = 0.439), time (*F*_(2,38)_ = 0.260, *P* = 0.772), and their interaction (*F*_(4,76)_ = 2.157, *P* = 0.082) on the 2-back task.

### Changes in arousal and pleasure levels induced by acute exercise

**Fig 4:**
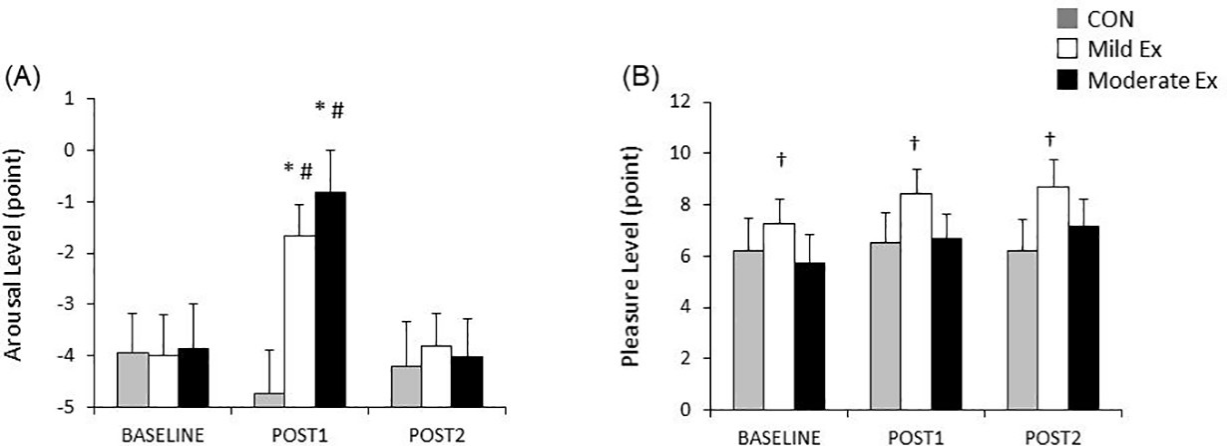
Changes in arousal and pleasure level for each session. Fig 4 shows the changes in arousal level (A) and pleasure level (B) for each session. The gray, white, and black bars indicate the CON, mild Ex, and moderate Ex conditions, respectively. The error bar indicates standard deviation. Arousal level increased after acute aerobic exercise, and was significantly higher in both exercise sessions than the CON session at post 1. Participants’ pleasure level in the mild Ex condition was significantly higher than the CON and moderate Ex conditions. *: *P* < 0.01 (baseline vs post 1), #: *P* < 0.001 (vs CON), †: *P* < 0.05 (vs other session).

Fig 4 shows the changes in arousal and pleasure levels for each session. There was a significant effect of session (*F*_(2,58)_ = 3.806, *P* < 0.05), time (*F*_(1.493,43.298)_ = 10.970, *P* < 0.001), and their interaction (*F*_(4,116)_ = 11.734, *P* < 0.001) on the arousal level. Post-hoc tests revealed that arousal levels increased from pre to post 1 (*P* < 0.001), and returned at post 2 in moderate Ex (*P* < 0.001). In mild Ex, the arousal level increased from pre to post 1 (*P* < 0.01), and it returned at post 2 (*P* < 0.01). Furthermore, arousal levels at post 1 in both exercise sessions were significantly higher than in the CON session (moderate Ex vs CON: *P* < 0.001, mild Ex vs CON: *P* < 0.001). There was a significant main effect of session on pleasure level (*F*_(1.675,48.572)_ = 8.950, *P* < 0.01). However, there was no significant effect of time (*F*_(1.500,43.509)_ = 2.413, *P* = 0.115) and no significant interaction between session and time (*F*_(4,116)_ = 1.479, *P* = 0.213). A post-hoc test revealed that pleasure level in mild Ex was higher than that of CON and moderate Ex (*P* < 0.05).

### Correlation between baseline WM function and the change in WM function

**Fig 5:**
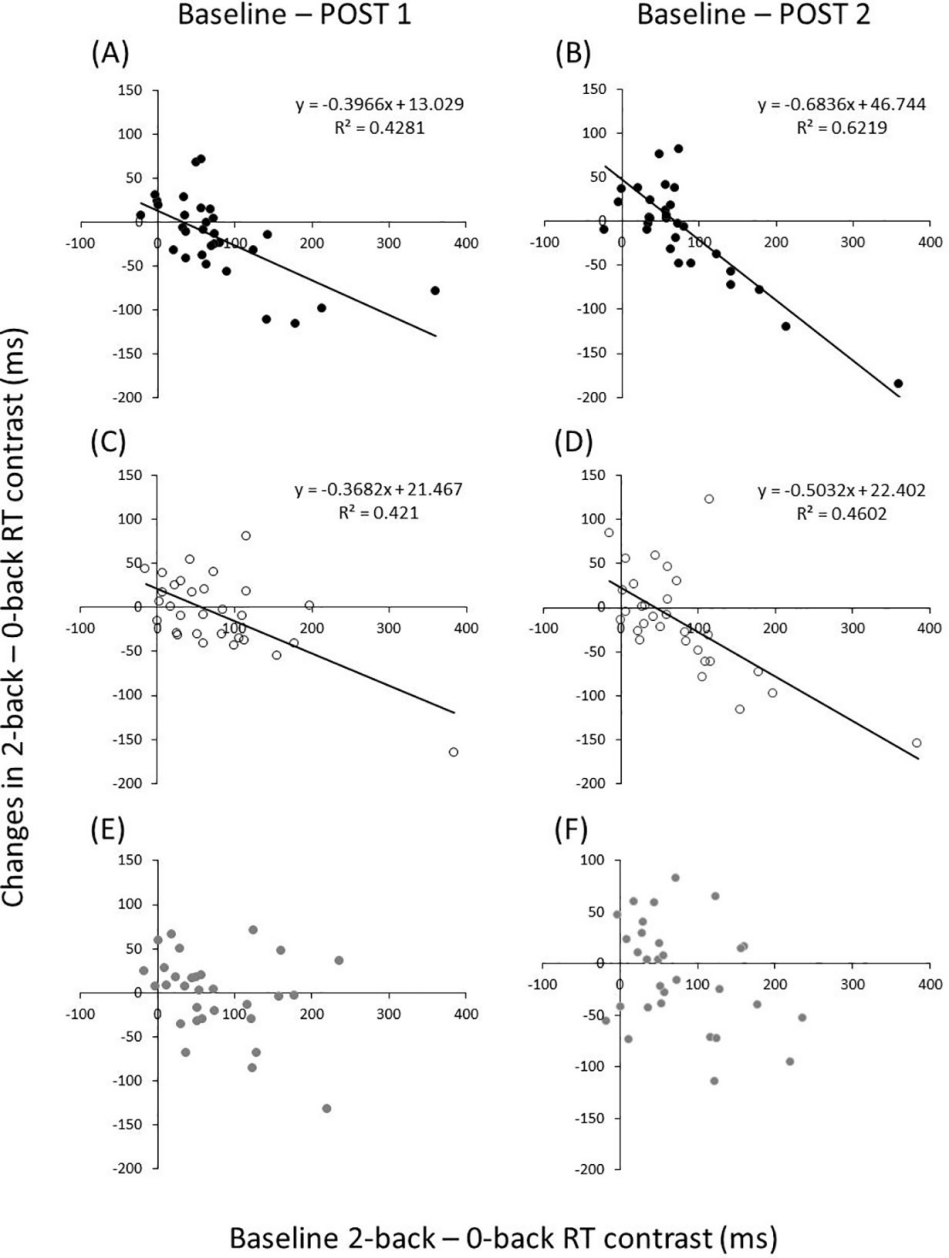
Correlation between baseline WM function and change of WM function by mild and moderate exercise. All scatterplots reveal the relationship between baseline WM function and change of WM function by acute exercise (A and B: moderate Ex, C and D: mild Ex, E and F: CON). Baseline WM function was significantly related to the change of WM function in acute exercise of both intensities.

Fig 5 and Table 2 show the correlation between baseline N-back task performance, the change in task performance of RT, and the error rate from baseline to post. In the moderate Ex session, a significantly negative correlation between baseline 2-back– 0-back RT contrast and the change in 2-back– 0-back contrast following exercise (post 1: r(30) = -0.65, *P* < 0.001; post 2: r(30) = -0.78, *P* < 0.001) was apparent (Fig 5A and 5B). Similarly, a significantly negative correlation between baseline 2-back– 0-back RT contrast and change in 2-back– 0-back RT contrast following exercise (post 1: r(30) = -0.64, *P* < 0.001; post 2: r(30) = -0.67, *P* < 0.001) in the mild Ex session (Fig 5C and 5D). All correlation coefficients were negative, meaning that 2-back– 0-back RT contrast shortened after exercise more in those with the lowest performance at baseline. Conversely, the correlation in the CON session was not significant (post 1: r(30) = -0.32, *P* = 0.082; post 2: r(30) = -0.33, *P* = 0.069) (Fig 5E and 5F).

**Table 2:** Relationships with baseline N-back task performance and changes in performance from baseline to post.

		Baseline–Post 1		Baseline–Post 2	
		r	*P*	r	*P*
0-back RT	CON	-0.28	0.121	-0.30	0.097
	Mild Ex	-0.30	0.098	-0.31	0.085
	Moderate Ex	-0.20	0.278	-0.09	0.622
0-back error rate	CON	-0.20	0.286	-0.29	0.109
	Mild Ex	-0.26	0.151	-0.27	0.145
	Moderate Ex	-0.30	0.102	-0.28	0.128
2-back RT	CON	-0.23	0.217	-0.34	0.061
	Mild Ex	-0.61	< 0.001*	-0.56	0.001*
	Moderate Ex	-0.54	0.001*	-0.67	< 0.001*
2-back error rate	CON	-0.31	0.091	-0.32	0.085
	Mild Ex	-0.32	0.081	-0.31	0.093
	Moderate Ex	-0.23	0.223	-0.28	0.128

* Significant correlation between baseline and post.

In the 0-back task, baseline RT did not correlate significantly with the change in RT following intervention in any of the sessions. However, there was a significant correlation between baseline 2-back RT and changes in 2-back RT in the mild and moderate Ex sessions.

The relationships between the baseline error rate and error rate after intervention were not significant in any of the sessions.

We compared the effect of acute aerobic exercise on WM performance between the high- and low-performance groups at baseline. A three-way repeated measures ANOVA showed the main effect of baseline performance, but no significant main effect and interactions (session: *F*_(2,56)_ = 0.030, *P* = 0.970; time: *F*_(2,56)_ = 3.069, *P* = 0.054; baseline performance: *F*_(1,28)_ = 148.931, *P* < 0.001; session × time: *F*_(2.660,74.476)_ = 0.874, *P* = 0.448; session × baseline performance: *F*_(2,56)_ = 0.402, *P* = 0.671; time × baseline performance: *F*_(2,56)_ = 3.088, *P* = 0.054; session × time × baseline performance: *F*_(4,112)_ = 0.953, *P* = 0.436).

## Discussion

We examined whether acute sessions of mild and moderate aerobic exercise improved WM function, and whether the improvements were dependent on baseline performance. The main findings were as follows. First, neither mild nor moderate aerobic exercise sessions of 10 min affected WM function. Second, the changes in 2-back– 0-back RT contrast correlated with baseline performance, irrespective of exercise intensity. These results suggest that the improvement of updating processing speed, seen after 10-min aerobic exercise sessions, depended on baseline performance and that this relationship was sustained for at least 15 min. This finding supported part of our hypothesis, that there is a relationship between the effect of acute aerobic exercise on WM that is dependent on baseline performance.

### The effect of mild and moderate acute exercise sessions on WM function

The results of this study showed that acute mild- or moderate-intensity exercise did not significantly improve WM function. Some previous studies have reported that acute moderate-intensity aerobic exercise improves WM. Pontifex et al. (2009) indicated that acute moderate-intensity aerobic exercise improved performance in a modified Sternberg test [18]. In addition, Weng et al. (2015) demonstrated that the accuracy of a 2-back task was improved more by acute moderate-intensity aerobic exercise than passive cycling, and that the improvement of accuracy was higher in the 2-back than the 0-back task [19]. Our findings were contrary to those of these previous studies, and the lack of a significant difference in WM function between the exercise and control sessions contradicted our hypothesis. However, the finding of a lack of an effect of acute exercise on WM function is not unique to this study. In the magnetic resonance imaging study reported by Li et al. (2014), acute aerobic exercise at a moderate intensity did not change WM function, even though changes in brain activity were observed [21]. Furthermore, Gothe et al. (2013) have suggested that N-back task performance did not change with acute moderate-intensity aerobic exercise [20]. As mentioned above, no consensus has yet been established regarding the effect of acute aerobic exercise on WM function. These previous studies investigated the effect of acute aerobic exercise on WM using only moderate intensity. Conversely, the present study was designed to examine the behavioral change of WM assessed in an N-back task following the cessation of both acute mild and moderate exercise, and to compare the effects of these two types of exercise intensity. The results showed that N-back task performance did not improve with exercised of either intensity. Therefore, our results suggest that 10 min of mild- or moderate-intensity aerobic exercise is insufficient to improve WM. The 0-back task requires sustained attention without WM-related function (e.g. maintenance and updating). Conversely, participants required maintenance and updating of information in addition to sustained attention during the 2-back task. It is thought that the 2-back– 0-back RT contrast is reflected in the time required for processing WM, including maintenance, manipulation, and updating. Because 2-back– 0-back RT did not improve, this demonstrates that neither acute mild- nor moderate-intensity aerobic exercise influenced the speed of processing of maintenance, manipulation, and updating.

One explanation for the lack of improvement of the N-back task performance was the influence of the task’s difficulty. Previous studies that showed improvement in WM after acute moderate aerobic exercise used relatively complicated WM tasks (modified Sternberg test and facial N-back task) [18, 19], and the RT of these tasks was more than 700 ms at baseline. However, the present study, and previous studies that did not show improved WM performance after exercise, used a simple N-back task (e.g. color or letter) [20, 21]. In fact, the RT of the 2-back task in these previous studies was 300–600 ms. Furthermore, the RT of the 2-back task in the present study was about 450 ms at baseline. Therefore, the effect of acute aerobic exercise might not be visible because of a ceiling effect in our study and these previous studies. Regarding another factor that may explain the lack of improvement in WM function after exercise, there is a possibility that the exercise effect varies according to the kind of cognitive function tested. In particular, changes in cognitive function after exercise may be greater for cognitive processes involved with inhibitory control as assessed using the Stroop task and the Go/No-Go task [24, 25, 32]. Our results showed that the change of WM function by each intensity of exercise varied greatly (Figs 3 and 5). These results suggest that there is large variability in WM improvement by acute aerobic exercise, and that this variability might be one of the factors accounting for why improvement following exercise was not seen.

As mentioned above, the 0-back task requires sustained attention but not WM demand [30]. According to our result of changes in the 0-back RT, acute mild and moderate aerobic exercise do not influence sustained attention. This finding is consistent with previous studies. Previous research examining the effects of acute exercise on cognitive performance has suggested a disproportionately larger benefit for tasks requiring greater amounts of executive control than for tasks with smaller executive requirements [33, 34]. Therefore, there is a possibility that improvement was not evident in the 0-back task because there was a low demand for cognitive processing.

One of the possible mechanisms for the effect of exercise on improved WM function is increased release of neurotransmitters. It has been shown that dopamine levels are strongly correlated with WM function [35, 36]. The relationship between dopamine levels and WM function is suggested to resemble an inverted U-shape [37]; optimum dopamine levels are beneficial for WM function. Previous studies have shown that exercise facilitates dopamine secretion [38, 39]. In addition, adrenaline and noradrenaline levels increase after acute exercise [40]. These neurotransmitters are associated with arousal and improvements in attention and memory [41]. The results of the TDMS in the current study showed that arousal level was increased by exercise. This finding is consistent with the result of Byun et al. [25], who reported that 10 min of mild-intensity exercise increased arousal levels. Interestingly, our results demonstrated that task performance was not improved but that arousal levels were increased. It is thought that one of the reasons for this discrepancy is the fact that different brain regions are involved in both WM and inhibitory control. While the prefrontal cortices play a crucial role in both of these functions [42], the activation of the posterior parietal cortex and the hippocampus are also important for WM [43–45]. The influence of arousal level might be different due to such differences in brain region activation.

### Inter-individual variability in the effect of acute exercise on WM

In this study, we detected a significant correlation between updating function at baseline and the change induced by acute exercise, irrespective of the intensity of the exercise session. Our previous study showed that there were high inter-individual differences in the improvement of spatial working memory following acute low-intensity aerobic exercise [46]. In addition, the activity of the right ventrolateral prefrontal cortex (PFC) during exercise was higher in participants who improved in spatial working memory after exercise than those who did not. Therefore, one possible mechanism of inter-individual differences by aerobic exercise is brain activity in the PFC during exercise.

Interestingly, the present results indicate that subjects with lower updating function at baseline could experience better improvements in updating function as a result of acute exercise. To our knowledge, only one previous study has shown that young adults with the lowest WM benefited from acute moderate aerobic exercise [22]. Unfortunately, features of the experimental setting of the exercise intervention might need to be considered. The intensity of the exercise is important when examining the effect of aerobic exercise on cognitive function, because changes in executive function resemble an inverted U-shaped response to different exercise intensities [32]. Thus, in this study, we controlled the exercise intensity according to the relative value of ${\dot{V}O}_{2\mathrm{peak}}$ in each subject, unlike previous studies that used self-paced exercise. Another important finding to consider is why inter-individual differences in updating function change produced by acute mild and moderate exercise appear to depend on baseline performance. One possible explanation is differences in exercise-induced cortical activity. Previous studies showed that cortical activity in the dorsolateral PFC (DLPFC) during the color word Stroop task significantly increased after 10 min of mild and moderate aerobic exercise, and reported similar improvements in inhibitory function [24, 25]. This may explain why exercise increased plasticity for tasks in the DLPFC; exercise may increase the levels of neurotrophic factors such as brain-derived neurotrophic factor and insulin-like growth factor 1 [8]. DLPFC plays a crucial role for WM updating [47, 48], and we speculate that increased DLPFC activity caused by mild and moderate exercise in this study largely explains the observed improvement in updating function. We were unable to find significant relationships with baseline error rate, or changes in it from baseline to post. Regarding this lack of relationships, the task difficulty may have been too easy for the subjects.

### Limitations

The present study has several limitations to consider. First, we included only young adults. WM is thought to decline gradually with age, and this decline induces cognitive impairment [49, 50]. It is possible that acute exercise is effective for improving WM in elderly people. Further studies should therefore perform similar analyses of elderly participants. Second, we did not investigate individual differences from a physiological perspective. Therefore, we were unable to clarify the mechanism through which differences in responses to acute exercise were expressed. Future studies will need to perform such physiological investigations.

## Conclusion

In the present study, we investigated whether acute mild and moderate aerobic exercise improves WM updating function. WM updating function was not significantly improved by either mild or moderate exercise. However, we were able to confirm, using correlation analysis, that the change in updating function caused by acute mild and moderate exercise was related to baseline performance.

## Supporting information

S1 File The Results of additional analysis. (DOCX)

S2 File Data set in all participants. (XLSX)
