# Supplementary material for: Inter-individual differences in working memory improvement after acute mild and moderate aerobic exercise
Source: PLoS One. 2018 Dec 31;13(12):e0210053. doi: 10.1371/journal.pone.0210053 (PMC6312311; doi:10.1371/journal.pone.0210053)
Supplement: S1 File — (DOCX) [file pone.0210053.s001.docx]

**Supporting Information**

We performed an additional analysis to investigate the relationships between the subjects’ cardiovascular capacity and baseline task performance, or changes in performance induced by acute aerobic exercise. Pearson’s correlation analysis was performed between subjects’ VO_2peak_ and N-back task performance (baseline RT, baseline error rate, change in RT, and change in error rate of 2-back, 0-back task, and 2-back – 0-back RT contrast in each condition).

The results showed no significant correlation between subjects’ VO_2peak_ and baseline, or changes in N-back task performance (*P* > 0.05) (Table S1).

Additionally, a mediation analysis was performed using the bootstrapping method, which is recommended for relatively small sample sizes. We examined (1) whether the experimental condition (independent variable) was associated with changes in updating performance (dependent variable); (2) whether the experimental condition (independent variable) was associated with baseline performance (mediator variable); (3) whether baseline performance (mediator variable) was associated with changes in updating performance (dependent variable); and (4) whether the prospective mediation effect was significant when the relationship between the experimental condition and changes in updating performance became significantly weaker (partial mediation) or insignificant (full mediation), after the inclusion of baseline performance. We could not find a significant relationship between the experimental condition (independent variable) and changes in updating performance (dependent variable) (*β* = -0.029, *P* = 0.814).

We changed each variable and performed the same analysis (dependent variable: changes in updating performance, independent variable: baseline performance, and mediator variable: changes in arousal level). We found a significant relationship between baseline performance (independent variable) and updating performance (dependent variable) (*β* = -0.647, *P* < 0.001). However, we could not find significant relationships between baseline performance (independent variable) and changes in arousal level (mediator variable) (*β* = 0.041, *P* = 0.738), or between changes in arousal level (mediator variable) and updating performance (dependent variable) (*β* = 0.020, *P* = 0.831). Therefore, changes in arousal level did not play a mediating role in the relationship between baseline performance and changes in updating performance (Table S2).

To examine the order effect, a one-way repeated-measures ANOVA was performed, including all sessions. We found a significant main effect of order of session in the 2-back – 0-back RT contrast, 2-back RT, 2-back error rate, and 0-back error rate. Post-hoc analysis revealed that the RT of the 3rd session was significantly shorter than that of the 1st session in the 2-back – 0-back RT contrast and 2-back RT. In addition, the 2-back error rate of the 3rd session was significantly lower than in the 1st session. The 0-back error rate was significantly lower in the 3rd session than in the 2nd session.

**Table S1. Correlation between subjects’ VO_2peak_ and N-back task performance**

|  | CON | | Mild Ex | | Mod Ex | |
| --- | --- | --- | --- | --- | --- | --- |
|  | r | *P* | r | *P* | r | *P* |
| Baseline |  |  |  |  |  |  |
| 0-back RT | 0.05 | 0.755 | 0.09 | 0.608 | 0.04 | 0.833 |
| 2-back RT | -0.12 | 0.498 | 0.01 | 0.953 | 0.02 | 0.926 |
| 2-back – 0-back RT contrast | -0.16 | 0.382 | -0.02 | 0.891 | 0.002 | 0.988 |
| 0-back error rate | -0.08 | 0.646 | -0.10 | 0.568 | -0.16 | 0.370 |
| 2-back error rate | -0.02 | 0.894 | 0.09 | 0.614 | -0.33 | 0.071 |
| Post 1 change |  |  |  |  |  |  |
| 0-back RT | 0.12 | 0.496 | 0.06 | 0.714 | -0.20 | 0.285 |
| 2-back RT | 0.08 | 0.642 | -0.16 | 0.393 | 0.02 | 0.878 |
| 2-back – 0-back RT contrast | 0.01 | 0.933 | -0.16 | 0.384 | 0.09 | 0.601 |
| 0-back error rate | -0.02 | 0.888 | -0.04 | 0.805 | 0.06 | 0.719 |
| 2-back error rate | -0.18 | 0.338 | -0.29 | 0.117 | 0.09 | 0.631 |
| Post 2 change |  |  |  |  |  |  |
| 0-back RT | 0.26 | 0.152 | -0.12 | 0.514 | 0.003 | 0.986 |
| 2-back RT | 0.08 | 0.655 | 0.002 | 0.988 | -0.10 | 0.573 |
| 2-back – 0-back RT contrast | 0.18 | 0.331 | 0.05 | 0.775 | -0.09 | 0.625 |
| 0-back error rate | -0.29 | 0.110 | -0.005 | 0.978 | 0.18 | 0.329 |
| 2-back error rate | -0.10 | 0.591 | 0.04 | 0.833 | 0.14 | 0.430 |

CON: control condition, Mild Ex: mild exercise condition, Mod Ex: moderate exercise condition.

**Table S2. Results of mediation analysis**

| Path | β | SE | *t*-value | *P*-value |
| --- | --- | --- | --- | --- |
| Experimental condition > change in performance (Total Effect) | -0.003 | 6.278 | -0.036 | 0.971 |
| Experimental condition > baseline performance | 0.039 | 11.278 | 0.321 | 0.749 |
| Baseline performance > change in performance | -0.648 | 0.067 | -6.959 | < 0.001 |
| Experimental condition > change in performance (Direct Effect) | -0.029 | 8.167 | -0.236 | 0.814 |
| 95% bootstrap CI | Lower | -13.204 | Upper | 7.400 |
|  |  |  |  |  |
| Baseline performance > change in performance (Total Effect) | -0.648 | 0.067 | -6.959 | < 0.001 |
| Baseline performance > change in arousal level | 0.041 | 0.006 | 0.336 | 0.738 |
| Change in arousal level > change in performance | 0.020 | 1.406 | 0.214 | 0.831 |
| Baseline performance > change in performance (Direct Effect) | -0.647 | 0.067 | -7.005 | < 0.001 |
| 95% bootstrap CI | Lower | -0.007 | Upper | 0.020 |

β: standardized regression coefficient, SE: standard error, CI: confidence interval.
